# Evaluation of the Potential Effects of Carnosine on Intestinal Ischemia/Reperfusion Injury in a Rat Model: A Lack of Significant Protective Effect

**DOI:** 10.7759/cureus.107891

**Published:** 2026-04-28

**Authors:** Ahmet R Iyiol

**Affiliations:** 1 General Surgery, Burdur State Hospital, Burdur, TUR

**Keywords:** carnosine, intestinal ischemia, mesenteric ischemia, oxidative stress, reperfusion injury

## Abstract

Background

Intestinal ischemia-reperfusion (I/R) injury represents a critical clinical condition characterized by oxidative stress, inflammatory activation, and impairment of intestinal mucosal integrity. Although restoration of blood flow is necessary to prevent irreversible tissue damage, reperfusion itself can aggravate injury through complex biochemical and cellular mechanisms. Despite improvements in surgical and intensive care management, effective pharmacological agents capable of reducing intestinal I/R injury remain limited. Carnosine, a naturally occurring dipeptide with antioxidant and anti-inflammatory properties, has been proposed as a potential protective agent in several experimental I/R models.

Objective

The aim of this study was to determine whether carnosine administration reduces histopathological mucosal injury scores and alters biochemical parameters in a rat model of intestinal I/R injury.

Methods

We allocated 30 adult Sprague-Dawley rats randomly into three groups (n = 10 each): control, I/R, and I/R + carnosine. Intestinal ischemia was established by clamping of the superior mesenteric artery (SMA) for 60 minutes, followed by 120 minutes of reperfusion. In the treatment group, carnosine (50 mg/kg, intraperitoneally) was administered immediately prior to the reperfusion phase. At the end of the reperfusion period, the animals were sacrificed, and terminal ileum tissue samples, together with intracardiac blood specimens, were collected. Histological injury was evaluated using the Chiu/Park mucosal injury scoring system. Serum malondialdehyde (MDA), blood urea nitrogen (BUN), creatinine (Cr), potassium (K), creatine kinase (CK), and phosphorus (P) levels were analyzed.

Results

Histopathological injury scores were significantly higher in both ischemic groups compared with the control group (p < 0.05). No statistically significant difference was observed between the I/R and I/R + carnosine groups (p > 0.05). Serum MDA levels were highest in the carnosine-treated group.

Conclusion

Carnosine did not demonstrate a protective effect against intestinal I/R injury under the conditions of this study. The elevated MDA levels observed in the treatment group further complicate the interpretation of its expected antioxidant effects. Further experimental studies are required to clarify the role of carnosine in intestinal I/R injury.

## Introduction

Acute mesenteric ischemia is a severe and often life-threatening clinical condition associated with considerable morbidity and mortality despite advances in diagnostic and therapeutic strategies [1]. Although restoration of blood flow is required to prevent irreversible intestinal damage, the reperfusion phase itself may aggravate tissue injury through complex molecular mechanisms [2].

Intestinal ischemia-reperfusion (I/R) injury develops through a multifactorial process involving oxidative stress, endothelial dysfunction, activation of inflammatory pathways, leukocyte recruitment, and impairment of epithelial barrier integrity. Excessive production of reactive oxygen species during reperfusion promotes lipid peroxidation and membrane injury, thereby contributing to structural and functional damage of the intestinal mucosa [3].

Carnosine (β-alanyl-L-histidine) is an endogenous dipeptide present in various tissues, including skeletal muscle and the nervous system. It has been reported to possess several biological properties such as antioxidant activity, free radical scavenging capacity, anti-glycation effects, and membrane-stabilizing functions [4]. Previous experimental studies have demonstrated the protective effects of carnosine in I/R injury involving different organs, including the liver, kidney, and brain [5-7].

A lipid peroxidation product named malondialdehyde (MDA) is widely utilized as a biochemical marker of oxidative stress in experimental models of I/R injury [3,8]. Increased levels of MDA reflect enhanced free radical-mediated membrane damage and oxidative cellular injury.

Despite these findings, evidence regarding the protective role of carnosine in intestinal I/R injury remains limited. Therefore, the present experimental study aimed to determine whether carnosine administration reduces histopathological mucosal injury scores and alters biochemical parameters in a rat model of intestinal I/R injury.

## Materials and methods

Study design and ethical approval

This experimental animal study was conducted at the Experimental Research Laboratory of Eskişehir Osmangazi University Faculty of Medicine, General Surgery Clinic, Medical and Surgical Research Center (T.I.C.A.M.), Eskişehir, Türkiye. Ethical approval was obtained from the Institutional Animal Ethics Committee (approval no. 287, September 5, 2012). The experiments were carried out between September 2012 and December 2013 in accordance with national guidelines for the care and use of laboratory animals.

Animals

Adult Sprague-Dawley rats with a mean body weight of 225 ± 25 g (range: 200-250 g) were included in the study. The animals were housed under controlled conditions with a 12-hour light/dark cycle and a room temperature of 22 ± 2°C. Food was withheld for 12 hours before surgery, while water was available ad libitum. Only healthy animals without clinical evidence of infection, gastrointestinal disease, or systemic illness were included. Animals with intraoperative complications, pre-existing illness, or death before completion of the protocol were excluded. Baseline characteristics of the animals are presented in Table 1.

**Table 1:** Baseline characteristics of experimental animals Adult Sprague-Dawley rats with a mean body weight of 225 ± 25 g (range: 200-250 g) were included. I/R: ischemia-reperfusion

Group	Number of rats	Body weight (g)
Control	10	225 ± 25
I/R	10	225 ± 25
I/R + carnosine	10	225 ± 25

Inclusion and exclusion criteria

Adult Sprague-Dawley rats with a mean body weight of 225 ± 25 g were included in the study. Only clinically healthy animals without signs of infection, gastrointestinal pathology, or systemic disease were enrolled. Animals that developed intraoperative complications, had evidence of pre-existing illness, or died before completion of the reperfusion period were excluded from the analysis.

Anesthesia and perioperative care

Anesthesia was induced with intramuscular ketamine (50 mg/kg; Ketalar®, Parke-Davis, Eczacıbaşı, Istanbul, Türkiye) and xylazine (10 mg/kg; Rompun®, Bayer AG, Leverkusen, Germany). The animals breathed spontaneously throughout the procedure. During the surgical procedure, body temperature was maintained at approximately 37°C using a heating lamp. Core body temperature was monitored intermittently and adjusted as needed to avoid hypothermia. Animals were placed on a temperature-controlled surface, and the distance of the heating lamp was adjusted to maintain normothermia throughout the procedure. To prevent dehydration, 10 mL of Ringer’s lactate solution was administered subcutaneously at the end of surgery.

Experimental groups

The rats were randomly assigned to three groups using a random number table to ensure unbiased allocation (n = 10 per group). This study was designed as a pilot (exploratory) experimental study, and no formal a priori power analysis was performed. Group 1 (Control): the superior mesenteric artery (SMA) was isolated without clamping; Group 2 (I/R): the SMA was clamped for 60 minutes, followed by 120 minutes of reperfusion; Group 3 (I/R + carnosine): the SMA was clamped for 60 minutes, and carnosine (50 mg/kg, intraperitoneally) was administered immediately before reperfusion; reperfusion was then continued for 120 minutes.

Surgical

**Figure 1:**
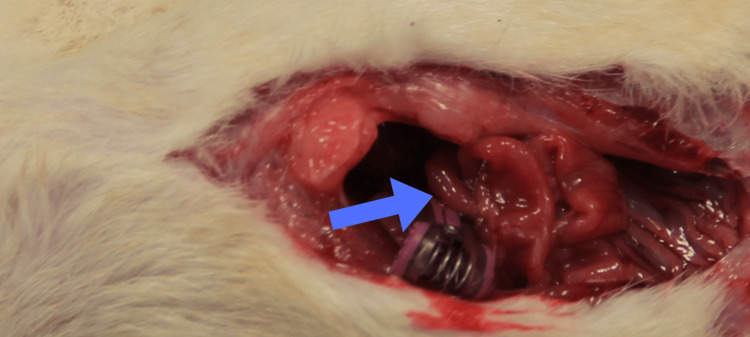
Isolation of the superior mesenteric artery (SMA) Representative intraoperative image demonstrating isolation of the SMA during midline laparotomy prior to vascular clamping for induction of intestinal ischemia (arrow).

Following adequate anesthesia, the abdomen was opened through a midline laparotomy under sterile conditions. The SMA was carefully isolated. The SMA was carefully isolated as shown in Figure 1.

Intestinal ischemia was induced by applying a microvascular clamp to the SMA immediately distal to its origin from the abdominal aorta. Successful ischemia was confirmed by pallor and absence of pulsation in the small intestine, cecum, and right colon.

After the ischemic period, the vascular clamp was removed, and intestinal reperfusion was allowed for the designated period. The abdominal incision was then closed in two layers using 3/0 polyglactin sutures (Vicryl®, Ethicon, England).

Histopathological evaluation

Terminal ileum specimens were fixed in buffered formalin, embedded in paraffin, sectioned at 4-5 μm, and stained with hematoxylin and eosin. Intestinal mucosal injury was graded using the Chiu/Park scoring system on a scale from 0 to 5, where grade 0 indicates normal mucosa and grade 5 indicates severe mucosal destruction [8]. All histopathological evaluations were conducted by a single experienced pathologist who was blinded to group allocation to reduce inter-observer variability and assessment bias.

Biochemical analysis

Biochemical parameters, including MDA, blood urea nitrogen (BUN), creatinine (Cr), potassium (K), creatine kinase (CK), and phosphorus (P) levels, were measured using commercially available assay kits according to the manufacturers’ instructions. MDA levels were determined by a spectrophotometric method based on thiobarbituric acid reactive substances (TBARS) using a commercial kit (Cayman Chemical, Ann Arbor, MI, USA). Serum BUN, Cr, K, CK, and P levels were analyzed using an automated biochemical analyzer (Roche Cobas c501, Roche Diagnostics, Mannheim, Germany) with corresponding commercial reagents.

Statistical analysis

Statistical analyses were performed using IBM SPSS Statistics for Windows, Version 15 (Released 2006; SPSS Inc., Chicago, IL, USA) and SigmaStat 3.1 (Systat Software Inc., San Jose, CA, USA). Data were expressed as mean ± standard deviation. One-way analysis of variance (ANOVA) was used for normally distributed data, while the Kruskal-Wallis test was used for non-normally distributed data. Histopathological scores, as ordinal data, were specifically analyzed using the Kruskal-Wallis test. When a significant difference was detected, post-hoc pairwise comparisons were performed using the Mann-Whitney U test with Bonferroni correction. A p-value <0.05 was considered statistically significant.

## Results

Histopathological findings

Histopathological examination of the terminal ileum specimens revealed normal mucosal architecture in all animals in the control group. According to the Chiu/Park classification, these specimens corresponded to Grade 0 injury, demonstrating intact villous structures and preserved epithelial lining without inflammatory infiltration.

In contrast, animals subjected to mesenteric ischemia followed by reperfusion exhibited varying degrees of mucosal injury. The histological alterations included epithelial lifting from the lamina propria, subepithelial edema, villous shortening, and focal mucosal erosion. In some specimens, inflammatory cell infiltration within the lamina propria was also observed.

The median histopathological injury scores were significantly higher in both the I/R and I/R + carnosine groups compared with the control group (p < 0.05). Post-hoc pairwise comparisons demonstrated significant differences between the control group and both ischemic groups, whereas no statistically significant difference was observed between the I/R and I/R + carnosine groups (p > 0.05).

Carnosine administration did not result in a statistically significant reduction in histopathological injury compared with the untreated I/R group.

The distribution of histopathological injury scores according to the Chiu/Park classification is summarized in Table 2.

**Table 2:** Histopathological injury scores according to the Chiu/Park classification Data were analyzed using the Kruskal-Wallis test. Post-hoc pairwise comparisons were performed using the Mann-Whitney U test with Bonferroni correction. * p < 0.05 vs. control group (I/R vs. control; I/R + carnosine vs. control). No statistically significant difference was observed between the I/R and I/R + carnosine groups. I/R: ischemia-reperfusion

Group	Median	25-75%	p-value
Control	0	0-0	-
I/R	2	0-5	<0.005*
I/R + carnosine	2	1-3	-

Biochemical findings

Serum BUN levels did not differ significantly among the experimental groups (p = 0.460). Although slightly higher median BUN levels were observed in the I/R group compared with the control group, these differences were not statistically significant.

BUN values for the experimental groups are presented in Table 3.

**Table 3:** Serum blood urea nitrogen (BUN) levels No statistically significant difference was observed. I/R: ischemia-reperfusion

Group	Median	25-75%	p-value
Control	25.55	23.90-31.10	-
I/R	27.85	25.20-31.80	0.460
I/R + carnosine	24.00	15.80-29.70	-

Creatinine (Cr)

Serum Cr levels were significantly elevated in both ischemic groups compared with the control group (p < 0.006). This finding may indicate systemic physiological effects associated with intestinal I/R injury. However, no statistically significant difference was observed between the untreated I/R group and the carnosine-treated group.

Cr values are summarized in Table 4.

**Table 4:** Serum creatinine levels *Statistically significant compared with the control group. I/R: ischemia-reperfusion

Group	Median	25-75%	p-value
Control	0.43	0.41-0.45	-
I/R	0.605	0.56-0.70	<0.006*
I/R + carnosine	0.61	0.48-0.69	-

Malondialdehyde (MDA)

Serum MDA levels were significantly increased in animals exposed to I/R injury compared with the control group (p < 0.05). The highest MDA levels were observed in the I/R + carnosine group. Post-hoc pairwise comparisons demonstrated that MDA levels in the I/R + carnosine group were significantly higher than both the control group and the untreated I/R group (p < 0.001). These findings indicate that carnosine administration was not associated with a reduction in systemic lipid peroxidation under the conditions of this study.

MDA values are shown in Table 5.

**Table 5:** Serum malondialdehyde (MDA) levels Data were analyzed using the Kruskal-Wallis test, followed by the Mann-Whitney U test with Bonferroni correction for pairwise comparisons. * p < 0.001 vs. control and I/R groups. I/R: ischemia-reperfusion

Group	Median	25-75%	p-value
Control	7.598	6.942-8.084	-
I/R	8.825	8.465-9.354	-
I/R + carnosine	13.926	12.169-14.783	<0.001*

Other biochemical parameters

No statistically significant differences were detected among the experimental groups in serum CK, K, or P levels.

## Discussion

Intestinal I/R injury is a major clinical condition associated with significant morbidity and mortality, particularly in conditions such as acute mesenteric ischemia, intestinal strangulation, and major vascular surgery [1]. Although restoration of blood flow is essential to preserve intestinal viability, the reperfusion phase paradoxically exacerbates tissue damage through complex biochemical and inflammatory mechanisms [2,3].

The pathophysiology of intestinal I/R injury is multifactorial and involves oxidative stress, inflammatory mediator activation, endothelial dysfunction, leukocyte adhesion, and disruption of intestinal epithelial integrity [3,9,10]. Reperfusion of previously ischemic tissue triggers reactive oxygen species generation and lipid peroxidation, leading to cellular and tissue injury [11,12]. These processes ultimately contribute to villous destruction and impairment of mucosal barrier function.

In the present study, mesenteric ischemia followed by reperfusion produced significant histopathological injury in the terminal ileum, confirming successful establishment of the experimental model. The observed morphological changes, including epithelial lifting, villous shortening, subepithelial edema, and inflammatory cell infiltration, are consistent with previously reported features of intestinal I/R injury.

Carnosine is an endogenous dipeptide with well-documented antioxidant and anti-inflammatory properties. However, in this study, carnosine administration did not result in a statistically significant reduction in histopathological injury compared with the untreated I/R group. Therefore, under the conditions of this study, no protective effect of carnosine could be demonstrated.

The biochemical findings further complicate interpretation. Serum MDA levels were highest in the I/R + carnosine group, which does not support the expected antioxidant effect. MDA is an indirect marker of lipid peroxidation, and TBARS-based spectrophotometric methods have limited specificity. In addition, systemic oxidative stress markers may not accurately reflect local intestinal tissue injury.

Although carnosine has established antioxidant properties, its biological effects may depend on experimental conditions such as dose, timing of administration, and the redox environment. The elevated MDA levels observed in this study may therefore reflect methodological limitations, systemic responses, or context-dependent biochemical effects rather than a clear antioxidant action. The lack of concordance between histopathological and biochemical findings supports a cautious interpretation.

In addition to local intestinal injury, systemic effects were also observed, as reflected by increased serum Cr levels in the ischemia groups. This finding suggests that intestinal I/R injury may contribute to remote organ dysfunction through mechanisms such as barrier disruption and systemic inflammatory responses.

Previous studies have demonstrated that impairment of mucosal barrier integrity during ischemia may facilitate translocation of bacterial products and inflammatory mediators into the systemic circulation, thereby contributing to remote organ dysfunction [13]. Recent advances have also highlighted the role of regulated cell death pathways, including apoptosis and ferroptosis, in the development of intestinal I/R injury. These findings emphasize the complex and multifactorial nature of reperfusion injury [14]. Future studies should incorporate more comprehensive biochemical and molecular analyses to better elucidate the mechanisms underlying intestinal I/R injury. In particular, the inclusion of tissue-based oxidative stress markers (e.g., superoxide dismutase, catalase, and glutathione-related enzymes), inflammatory cytokines (e.g., tumor necrosis factor-alpha (TNF-α) and interleukin 6 (IL-6)), apoptosis indicators, and intestinal barrier integrity markers would provide a more detailed understanding of the potential effects of carnosine.

From a clinical perspective, pharmacological agents targeting oxidative stress and inflammatory pathways during reperfusion may offer therapeutic benefit; however, the findings of this study indicate that carnosine does not provide such benefit under the conditions tested.

Limitations

This study has several limitations. First, the sample size was relatively small, and no formal a priori power analysis was performed; therefore, the study may have been underpowered to detect modest treatment effects. Second, only a single dose and administration timing of carnosine were evaluated, which limits conclusions regarding optimal therapeutic conditions. Third, oxidative stress assessment was based primarily on serum MDA levels, which may not accurately represent local intestinal oxidative injury and are subject to methodological limitations. Fourth, although histopathological evaluation was performed in a blinded manner, inter-observer variability was not assessed. Finally, as this is an experimental animal study, the findings cannot be directly extrapolated to clinical practice.

Taken together, these limitations highlight the need for further experimental studies incorporating larger sample sizes, different dosing regimens, and more specific oxidative stress markers to better clarify the potential role of carnosine in intestinal I/R injury.

## Conclusions

Intestinal I/R injury is a complex pathological process characterized by oxidative stress, inflammatory activation, and significant intestinal mucosal damage. In the present experimental rat model, mesenteric ischemia followed by reperfusion resulted in substantial histopathological injury and increased oxidative stress markers. Carnosine administration did not produce a statistically significant reduction in mucosal injury compared with untreated I/R. In addition, the elevated serum MDA levels observed in the carnosine-treated group do not support the expected antioxidant effect under the conditions of this study. These findings indicate that carnosine did not demonstrate a protective effect against intestinal I/R injury.

Further experimental studies incorporating larger sample sizes, different dosing strategies, earlier administration protocols, and combined antioxidant approaches are required to better clarify the therapeutic potential of carnosine in intestinal I/R injury. In addition, future studies should include more comprehensive biochemical and molecular analyses, particularly tissue-based oxidative stress markers (e.g., superoxide dismutase, catalase, and glutathione-related enzymes), inflammatory cytokines (e.g., TNF-α and IL-6), apoptosis indicators, and intestinal barrier integrity markers.
